# Clinical Progress of PD-1/L1 Inhibitors in Breast Cancer Immunotherapy

**DOI:** 10.3389/fonc.2021.724424

**Published:** 2022-01-06

**Authors:** Fei Chen, Naifei Chen, Yangyang Gao, Lin Jia, Zheng Lyu, Jiuwei Cui

**Affiliations:** Cancer Center, the First Hospital of Jilin University, Changchun, China

**Keywords:** breast cancer, immunotherapy, immune checkpoint inhibitor, PD-1, PD-L1

## Abstract

Breast cancer is a major killer of women’s health worldwide. While breast cancer is thought to have lower immunogenicity compared with other solid tumors, combination therapy is able to improve the immunogenicity of the tumor and sensitize breast cancer cells to immunotherapy. Immunotherapy represented by immune checkpoint inhibitors (ICIs) has been largely explored in the field of breast cancer, including both early and advanced disease. Immunotherapy for triple-negative breast cancer (TNBC) has been the most studied, and the PD-L1 inhibitor atezolizumab combined with nab-paclitaxel has been used in the first-line treatment of TNBC. Immunotherapeutic data for human epidermal growth factor receptor-positive and hormone receptor-positive breast cancer are also accumulating. This review summarizes the clinical trial data of ICIs or ICI-containing therapies in different types and stages of breast cancer.

## 1 Introduction

Immunotherapy represented by immune checkpoint inhibitors (ICIs) has become an important strategy for the treatment of malignant tumors. With the increase in the indications of programmed cell death receptor 1 (PD-1) and programmed cell death 1 ligand 1 (PD-L1) inhibitors, the treatment pattern of many solid tumors has gradually changed (1). However, the development of immunotherapy in breast cancer is relatively slow. Breast cancer (BC) is traditionally considered to be poorly immunogenic. Due to the heterogeneity of molecular subtypes of breast cancer, the immune microenvironment of each subtype is discrepant (2, 3), which is one of the challenges of breast cancer immunotherapy. The further research of tumor immune microenvironment brings new opportunities for immunotherapy of this disease. Based on the Impassion130 study, PD-L1 inhibitor atezolizumab combined with nab-paclitaxel has been approved in the first-line treatment of triple-negative breast cancer (TNBC) (4, 5), which opens up a new window for the treatment of advanced TNBC. The exploration of ICI monotherapy and combination therapy involves multiple disease stages of TNBC. Immunotherapy has also been increasingly explored in human epidermal growth factor receptor 2 (HER2)-positive and hormone receptor-positive breast cancer. Immunotherapy may become a new treatment paradigm for breast cancer. As the existence of heterogeneity in the tumor microenvironment of different molecular types of breast cancer, and the inconsistent efficacy of immunotherapy, we reviewed the current clinical trial evidence for breast cancer immunotherapy according to molecular subtypes.

## 2 PD-1/L1 Inhibitors for Triple-Negative Breast Cancer

TNBC accounts for about 15%–20% of all breast cancers (6). Due to the lack of hormone receptor and HER2 expression, chemotherapy has been the mainstay treatments for TNBC for many years (7). However, suboptimal survival and tolerance of chemotherapy impels the development of novel strategies for treating this difficult-to-treat disease (8). There are several lines of supporting evidence for the potential of immunotherapy in TNBC. High expression of the immunomodulatory genes is associated with better outcomes for patients with TNBC (9). Higher enrichment of tumor-infiltrating lymphocytes (TILs) has been shown to be a prognostic predictor in TNBC (10–12). TNBC cells harbored higher level of PD-L1 expression than non-TNBC cells (13). Based on these rationale, immune checkpoint inhibitors (ICIs) represented by PD-1/L1 inhibitors are increasingly being explored for the treatment of early-stage TNBC and advanced-stage TNBC.

### 2.1 Neoadjuvant Therapy

#### 2.1.1 Combination of Immunotherapy and Chemotherapy

The phase III KEYNOTE-522 study (14) randomized 1,174 early TNBC patients to the neoadjuvant chemotherapy (carboplatin plus paclitaxel and sequential doxorubicin/epirubicin plus cyclophosphamide) combined with pembrolizumab, a PD-1 inhibitor, followed by adjuvant pembrolizumab, or chemotherapy alone, followed by adjuvant placebo. The results showed that the pathological complete response (pCR) rate was significantly higher in the pembrolizumab plus chemotherapy group (64.8% vs. 51.2%) for the overall population. Moreover, patients with PD-L1 expression, which was assessed by PD-L1 22C3 pharmDx assay, and positive lymph nodes benefited more from pembrolizumab. In terms of event-free survival (EFS), the 18-month EFS rates were 91.3% and 85.3%, respectively, and the hazard ratio (HR) for EFS was supportive of pembrolizumab addition (HR 0.63, 95% confidence interval (CI) 0.43–0.93). The phase III NeoTRIPaPDL1 study (15) randomized 280 patients with early TNBC to atezolizumab (anti-PD-L1) plus carboplatin/nab-paclitaxel arm or placebo plus carboplatin/nab-paclitaxel arm. The results showed that the pCR was not significantly different in neither the overall population (43.5% vs. 40.8%) nor the PD-L1-positive (determined by VENTANA PD-L1 SP142 assay) population (51.9% vs. 48.0%). IMpassion031 (16), a phase III trial, showed that atezolizumab plus nab-paclitaxel and sequential doxorubicin/cyclophosphamide increased pCR rate to 58%, compared with 41% in the chemotherapy group. In the PD-L1-positive subgroup (identified by VENTANA SP142 assay), pCR rate was 20% higher in the atezolizumab group (69% vs. 49%). Treatment-related grades 3–4 AEs were balanced (57% vs. 53%) and treatment-related serious AEs were 23% and 16%, respectively. PD-L1 inhibitor durvalumab addition to sequential taxane–anthracycline chemotherapy was investigated in GeparNuevo (17). In this phase II trial, 117 patients were randomized to the window-phase durvalumab group (durvalumab was administered 2 weeks before the beginning of nab-paclitaxel). The pCR of these patients was 61.0%, compared with 41.4% for the placebo cohorts (OR 2.22, 95% CI 1.06–4.64). While for the nonwindow dosing cohort (*n* = 57), no advantage of durvalumab was observed over chemotherapy.

#### 2.1.2 Combination of Immunotherapy and Targeted Therapy

The phase II I-SPY 2 trial tested the efficacy and safety of durvalumab plus PARP inhibitor olaparib and paclitaxel compared with paclitaxel alone in the neoadjuvant setting of TNBC treatment (18). In 21 TNBC patients, the estimated pCR rate was 47% with the combination subgroup and 27% with chemotherapy alone subgroup. Further biomarker analysis showed that low CD3/CD8 gene signature ratio, high macrophage/Tc-class 2 ratio, and high proliferation signatures were associated with a higher pCR in the combination arm.

### 2.2 Maintenance Therapy

#### 2.2.1 Combination of Immunotherapy and Chemotherapy

A phase II RCT named SAFIR02-BREAST IMMUNO compared durvalumab with chemotherapy (paclitaxel, capecitabine, and FEC) in the maintenance therapy for HER2-negative metastatic breast cancer (19). Patients with disease that did not progress after 6 to 8 cycles of first-line or second-line chemotherapy were included. In the exploratory TNBC subgroup analysis, the OS was significantly improved in the durvalumab arm (21 months vs. 14 months, HR 0.54, 95% CI 0.30–0.97); PD-L1-positive (detected by VENTANA PD-L1 SP142 assay) patients benefited more from durvalumab administration than from chemotherapy (HR 0.37, 95% CI 0.12–1.13), while PD-L1-negative patients did not benefit much (HR 0.49, 95% CI 0.18–1.34). In addition, TNBC patients with CD274 gain/amplification could benefit from durvalumab over chemotherapy in OS (HR 0.18, 95% CI 0.05–0.71), compared with patients with CD274 normal/loss (HR 1.12, 95% CI 0.42–2.99).

### 2.3 First-Line Therapy

#### 2.3.1 Immunotherapy Alone

ICI monotherapy was firstly explored in advanced-stage TNBC treatment. In cohort B of the international phase II KEYNOTE-086 study, pembrolizumab as first-line treatment for metastatic TNBC patients with tumor PD-L1 combined positive score (CPS) ≥1 was evaluated with PD-L1 22C3 pharmDx assay. In this cohort, 84 untreated patients with PD-L1-expressing metastatic TNBC received pembrolizumab 200 mg/3 weeks for up to 2 years. The ORR, PFS, and OS were 21.4%, 2.1 months, and 18 months, respectively. As for grade 3 or higher AEs, the incidence rate was 9.5% (20). The PCD4989g study, an open-label, multicenter phase 1a study, evaluated atezolizumab monotherapy in advanced solid and hematologic malignancies, which enrolled 116 metastatic TNBC patients. In 21 first-line patients, the ORR was 24% and median OS was 17.6 months. Of 116 patients in all lines, grade 3 or above AEs accounted for 21%. Patients with PD-L1 ≥1% had higher ORR and longer OS, and PD-L1 ≥10% was an independent predictor of better response and survival, with VENTANA PD-L1 SP142 assay being used for quantifying PD-L1 expression (21).

#### 2.3.2 Combination of Immunotherapy and Chemotherapy

Chemotherapy is demonstrated to be capable of enhancing tumor immunogenicity and T-cell-dependent antitumor response (22). Several studies of PD-1/L1 antibodies combined with chemotherapeutic agents have been performed in the first-line treatment of TNBC. The IMpassion130 study assessed the efficacy of atezolizumab in combination with nab-paclitaxel in patients with unresectable, locally advanced, or metastatic TNBC (23, 24). In the overall intention-to-treat (ITT) population, the combination of atezolizumab and nab-paclitaxel resulted in a significant improvement in median PFS (7.2 months vs. 5.5 months; HR 0.80, 95% CI 0.69–0.92); however, no statistically significant increment in median OS was observed in the atezolizumab arm (21 months vs. 18.7 months; HR 0.86, 95% CI 0.72–1.02). The ORR in atezolizumab group was 56.0%, versus that of 45.9% in the nab-paclitaxel group. Furthermore, for the PD-L1 (evaluated by VENTANA PD-L1 SP142 assay) positive subgroup (41% of all patients), the median PFS of combination group and chemotherapy group were 7.5 months and 5.0 months, respectively (HR 0.62, 95% CI 0.49–0.78); the median OS was significantly prolonged in the combination group (25 months vs. 18 months, HR 0.71, 95% CI 0.54–0.94), though that could not be formally concluded owing to the prespecified statistical testing hierarchy. While, for the PD-L1-negative patients, there was no difference in OS between the two groups (19.7 months vs. 19.6 months, HR 0.97; 95% CI 0.78–1.20). Patients receiving atezolizumab experienced more grade 3 or higher AEs (40.4% vs. 30.7%). In March 2019, the FDA approved atezolizumab plus nab-paclitaxel for first-line treatment of locally advanced or metastatic TNBC with PD-L1 ≥1%.However, atezolizumab plus paclitaxel, assessed in IMpassion131 study, failed to improve PFS or OS compared with paclitaxel alone (25). The phase III IMpassion132 study of gemcitabine plus carboplatin/capecitabine with or without atezolizumab as the first-line therapy of TNBC is ongoing (NCT03371017). In the phase III KEYNOTE-355 study (26, 27), pembrolizumab combined with chemotherapy (nab-paclitaxel; paclitaxel; or gemcitabine/carboplatin) as the first-line treatment of advanced TNBC mainly benefited patients with ≥10% PD-L1 expression (detected by PD-L1 22C3 pharmDx assay) disease, with a median PFS of 9.7 months in combination group versus 5.6 months in chemotherapy group (HR 0.66, 95% CI 0.50–0.88).With respect to grades 3–5 AEs, 68.1% patients experienced that in the combination group (2 deaths), in contrast with 66.9% in the chemotherapy group (0 death). However, more patients suffered grades 3–4 immune-related AEs and infusion reactions in the immunochemotherapy group (5.5% vs. 0%).

#### 2.3.3 Combination of Immunotherapy and Targeted Therapy

Hyperactivation of the PI3K/AKT pathway, resulted from the downregulation of *PTEN* gene, is one of the dominant mechanisms of tumor progression (28). Agents targeting the PI3K/AKT pathway may augment the antitumor adaptive immune responses (29). Bases on this rationale, a phase 1b study of combining AKT inhibitor ipatasertib, atezolizumab, and chemotherapy (paclitaxel or nab-paclitaxel) as first-line treatment for locally advanced or metastatic TNBC was performed. The results showed that the ORR reached 54%, with manageable toxicity (30). Antiangiogenic therapy is shown to have a synergistic antitumor effect with anti-PD-1 therapy (31). The phase II WJOG9917B NEWBEAT study evaluated the triple combination of PD-1 inhibitor nivolumab, bevacizumab, and paclitaxel in the first-line treatment for patients with TNBC (*n* = 18, 32%) or hormone receptor-positive breast cancer (*n* = 39, 68%) (32). This combination therapy led to an ORR of 83.3% in patients with TNBC, which demonstrated promising synergistic efficacy of VEGF inhibitor addition to immunochemotherapy. Another phase II trial explored PD-1 inhibitor camrelizumab (SHR-1210) combined with apatinib for advanced TNBC patients (*n* = 34). The results showed that apatinib continuous dosing group (d1–d14 administration) had an ORR and DCR of 47.4% and 68.4%, respectively. In apatinib intermittent dosing group (d1–d7 administration), there was no confirmed ORR, with a DCR of 44.4% and a PFS of 2 months (33). TNBC usually has upregulated MAPK pathway and increased sensitivity to MEK inhibition. MEK inhibitor increases the levels of effector CD8^+^ T cells in tumors and synergizes with anti-PD-L1 blockade (34). The cohort 1 of COLET study showed that MEK1/2 inhibitor cobimetinib plus paclitaxel could enhance antitumor effects for the first-line treatment of TNBC (35). IMpassion130 illustrated that the combination of atezolizumab and nab-paclitaxel as first-line treatment is effective for TNBC patients (24). Therefore, the cohort 2 of COLET evaluated the efficacy and safety of atezolizumab plus cobimetinib plus nab-paclitaxel or paclitaxel as first-line treatment of locally advanced or metastatic TNBC (36). The results showed that the ORRs were similar between the nab-paclitaxel and paclitaxel arms (29% vs. 34%). Patients with PD-L1-positive disease had numerically higher ORR (44%) and 6-month PFS rate. The safety profile of combined treatments was consistent with the known individual safety profiles.

### 2.4 Second-Line or Later Therapy

#### 2.4.1 Immunotherapy Alone

In the TNBC cohort of the phase 1b KEYNOTE-012 study, pembrolizumab as the first-line or later treatment yielded an ORR of 18.5%, PFS of 1.9 months, OS of 10.2 months, and ≥grade 3 AEs of 18.8% (37, 38). Cohort A of KEYNOTE-086 tested the efficacy of pembrolizumab as second-line or later therapy of metastatic TNBC. In the total patients (*n* = 170), the ORR was 5.3%, and PFS and OS were 2.0 months and 9.0 months, respectively; grade 3 or above AEs were 12.9%. The PD-L1 ≥1% population derived similar benefits as the overall patients, with an ORR of 5.7%, PFS of 2.0 months and OS of 8.8 months (39). KEYNOTE-119 compared pembrolizumab with chemotherapy (capecitabine, eribulin, gemcitabine, and vinorelbine) in second-line or third-line setting for metastatic TNBC patients (40). The results showed that pembrolizumab did not present superior efficacy over chemotherapy, except the exploratory subgroup who had PD-L1 CPS of 20 or higher, with OS being 14.9 months versus 12.5 months (HR 0.58, 95% CI 0.38–0.88). Moreover, the grades 3–5 AEs were lower in the pembrolizumab group (14% vs. 36%). The phase 1b JAVELIN study evaluated avelumab, an PD-L1 inhibitor, in patients with metastatic breast cancer who had received a median of three prior cytotoxic therapies. In the TNBC cohort (*n* = 58), the ORR was 5.2% in PD-L1 nonselected patients, and patients with PD-L1-positive (assessed by PD-L1 73-10 pharmDx assay) disease had an ORR of 22.2% (41), which further clarified the PD-L1 prevalence is an important predictor of immunotherapy. Additionally, the PCD4989g study showed that atezolizumab monotherapy yielded an ORR of 11% and an OS of 7.3 months for TNBC patients in second-line and beyond setting (21).

#### 2.4.2 Induction Therapy and Sequential Immunotherapy

To date, the timing of immunotherapy dosing remains to be further explored and studied. The TONIC study is a phase II RCT of nivolumab after induction therapy for metastatic TNBC (42). Patients were randomized into induction therapy groups (radiotherapy, doxorubicin, cyclophosphamide, cisplatin) or no induction therapy groups, followed by sequential nivolumab. The results showed that the ORR was 20% in all-line patients, including 23%, 45%, and 32% ORR for 1, 2, and later lines of patients, respectively. The ORR was 8%, 35%, 8%, and 23% in the radiotherapy, doxorubicin, cyclophosphamide, and cisplatin induction groups, respectively, compared with 17% in the noninduction group. Therefore, induction therapy with doxorubicin and cisplatin could improve the sensitivity of TNBC to immunotherapy. Future randomized controlled studies with larger samples comparing the efficacy differences between simultaneous versus sequential administration are expected.

#### 2.4.3 Combination of Immunotherapy and PARP Inhibitor

DNA repair deficiency in cancer cells contributes to immunogenic neoantigens accumulation, and PARP blockade can upregulate PD-L1 expression in breast cancer cells (43). Thereby, the combined treatment of PARP inhibitor and PD-1/L1 inhibitor is a potential strategy to treat breast cancer. The phase II TOPACIO/KEYNOTE-162 trial showed promising antitumor activity of PARP inhibitor niraparib plus pembrolizumab in patients who had received a median of 1 prior line of therapy (0–3) in the metastatic setting (44). The ORR was 21% and DCR was 49%. In 15 *BRCA*-mutated patients, the ORR, DCR, and PFS was 47%, 80%, and 8.3 months, respectively, which were both greater than that of patients with wild-type *BRCA* (11%, 33%, 2.1 months). Furthermore, patients with PD-L1-positive (examined by PD-L1 22C3 pharmDx assay) cancers responded better than those with PD-L1-negative ones (32% vs. 8%). The most common grade 3 or higher AEs were anemia (18%), thrombocytopenia (15%), and fatigue (7%). The breast cancer cohort of the MEDIOLA study (open-label, multicenter, phase I/II) explored the efficacy of PARP inhibitor olaparib combined with durvalumab in advanced *BRCA*-mutated HER2-negative metastatic breast cancer, and the results showed that the ORR reached 63.3%, and the PFS and OS were 8.2 months and 21.5 months, respectively (45).

#### 2.4.4 Combination of Immunotherapy and Immunomodulator

Imprime PGG (Imprime) is a novel immune agonist that activates the innate immune system to reregulate the immunosuppressive tumor microenvironment, activate antigen-presenting cells, and stimulate antigen-specific T-cell activation (46, 47). Preclinical studies showed that Imprime significantly enhanced the antitumor efficacy of ICIs (48). The phase II IMPRIME1 study investigated Imprime addition to pembrolizumab for second-line and later TNBC patients (49). The ORR was 15.9% and PFS was 16.4 months. The 12-month and 18-month OS rates were 57.6% and 36.7%, respectively. Notably, the study observed an ORR of 50% and an OS of 17.1 months in 12 patients who initially had hormone receptor-positive disease but converted to TNBC after endocrine therapy. Grades 3–4 AEs occurred in 6.8% of patients. These data validate the preclinical findings and provide clinical evidence for the immunomodulator-ICI combination in the treatment of TNBC. Large randomized controlled studies are needed to further clarify the advantages of this novel therapy.

Taken together, ICIs have been assessed in multiple settings for TNBC treatment. Two studies of pembrolizumab and atezolizumab in the adjuvant treatment of TNBC (SWOG S1418/NRG BR006, IMpassion030) are recruited (50, 51). The role of immunotherapy in the neoadjuvant treatment of TNBC still needs to be verified by updated EFS and OS data. We could see that the subset of TNBC patients benefited from immunotherapy mainly were these with PD-L1 expression ≥1%. Although the predictive threshold of PD-L1 varies across studies, in general, the benefit may be more pronounced with higher levels of PD-L1 expression. TMB is another predictor of ICI efficacy in TNBC patients. Furthermore, applying immunotherapy at earlier lines was associated with higher response rate. ICI is superior to chemotherapy in the maintenance treatment of metastatic TNBC. ICI monotherapy leads to suboptimal tumor response and patients’ survival, and its combination with chemotherapy and (or) targeted therapy is more effective but accompanied by increased incidence of AEs. These findings suggest a meaningful clinical benefits of ICI addition to standard chemotherapy and (or) targeted agents for the treatment of locally advanced or metastatic TNBC. However, several questions such as optimal chemotherapeutic partner and sequence of administration and difference between anti-PD-1 and anti-PD-L1 inhibitors remain unknown. A phase II trial of pembrolizumab versus nivolumab versus atezolizumab, all combined with chemotherapy, for metastatic TNBC treatment is ongoing (NCT03952325). Additionally, according to transcriptomic profile, TNBC can be classified into luminal androgen receptor, immunomodulatory, basal-like immune-suppressed, and mesenchymal-like subtypes (52). Immunomodulatory TNBC is deemed to be sensitive to immune checkpoint blockade therapy (52). However, there are no data on the difference in the responsiveness of TNBC subtypes to immunotherapy. Further studies of subtypes are needed to select benefited population and achieve precise immunotherapy for TNBC.

## 3 PD-1/L1 Inhibitors for HER2-Positive Breast Cancer

Previous studies indicated that substantial quantities of lymphocytic infiltrate in the tumor stroma is associated with achieving a pathological complete response and having improved survival in patients with HER2-positive breast cancer (53–56). High expression of PD-1/L1 and other checkpoint molecules was observed in TILs (56, 57). Trastuzumab, an antibody of HER2, can exert antitumor immune effects through antibody-dependent cellular cytotoxicity and phagocytosis and complement-dependent cytotoxicity (58). Preclinical studies discovered that the combination of ICIs and trastuzumab could reverse trastuzumab resistance (59). Based on these evidences, several clinical trials evaluated the value of ICIs combined with anti-HER2 treatment in HER2-positive advanced breast cancer.

### 3.1 Second-Line or Later Therapy

#### 3.1.1 Combination of Immunotherapy and Anti-HER2 Treatment

The phase Ib-II PANACEA study investigated the efficacy and safety of pembrolizumab plus trastuzumab in advanced HER2-positive breast cancer resistant to previous multiple lines of trastuzumab-containing therapies (60). Its phase II results showed that in HER2-positive, advanced, heavily pretreated breast cancer patients, the ORR of PD-L1-positive patients (*n* = 40, selected by PD-L1 22C3 pharmDx assay) was 15%, the median PFS was 2.7 months, the estimated 6-month PFS rate was 25%, and the 12-month PFS rate was 12%; the median OS has not been reached, and the 6-month and 12-month OS rates were estimated to be 87% and 65%, respectively. However, for PD-L1-negative patients (*n* = 12), no one achieved objective response or disease control, the median PFS was 2.5 months, and the estimated 6-month and 12-month PFS rates were 13% and 0%, respectively; the median OS was 7.0 months, 6-month OS rate was estimated to be 64%, and 1-year OS rate was estimated to be merely 12%. Moreover, patients achieving response and disease control had more TILs in metastatic lesions. In terms of AEs, 29% patients experienced treatment-related grades 3–5 AEs. The phase Ib CCTG IND.229 study tested the combination of durvalumab and trastuzumab in HER2-positive metastatic breast cancer patients who pretreated with trastuzumab, pertuzumab, T-DM1, and lapatinib (61). All enrolled 15 patients had PD-L1-negative (assessed by VENTANA PD-L1 SP263 assay) disease, and evaluable pretreatment and on‐treatment tumor biopsies (*n* = 5) had sparse CD8 cell infiltration. The results showed that none of these patients achieved response and their long-term survival was also disappointing. Another phase Ib study explored the safety and efficacy of pembrolizumab in combination with T-DM1 in patients with HER2-positive metastatic breast cancer previously treated with trastuzumab, pertuzumab, and paclitaxel (62). The results showed that the overall ORR was 20%, PFS was 9.6 months, and DOR was 10.1 months. Additionally, no correlation between the expression level of PD-L1 (examined by PD-L1 22C3 pharmDx assay) or the proportion of TILs and efficacy was observed. The randomized phase II KATE2 study compared the efficacy of T-DM1 combined with atezolizumab with T-DM1 alone in the second-line treatment of HER2-positive breast cancer (63). The results showed that there was no significant difference in PFS between the T-DM1 plus atezolizumab group and the T-DM1 plus placebo group (8.2 months vs. 6.8 months, HR 0.82, 95% CI 0.55–1.23), neither in the PD-L1-positive (diagnosed by VENTANA PD-L1 SP142 assay) subgroup (8.5 months vs. 4.1 months, HR 0.60, 95% CI 0.32–1.11) nor in the PD-L1-negative subgroup (6.8 months vs. 8.2 months, HR 1.02, 95% CI 0.60–1.74). In the PD-L1-positive subgroup, the ORR was higher in the atezolizumab group (54% vs. 33%). However, in PD-L1-negative patients, the ORR was inferior in the combination group (39% vs. 50%). The OS curves of the two groups separated after 1 year of follow-up, and the median OS has not been reached.

From the above studies, the efficacy of PD-1/L1 inhibitors combined with anti-HER2 therapy for heavily pretreated HER2-positive advanced breast cancer seems to be unsatisfactory. There are no reliable markers that can accurately predict the benefited population. Notably, these studies had small sample sizes, included patients who had heavy tumor burden and progressed on multiple prior anti-HER2 therapies, which possibly explained the suboptimal results. In the future, it may be worth to assess the tumor microenvironment, explore practical immune-related predictive biomarkers of efficacy, and apply ICIs combined with anti-HER2 therapy in early-stage or first-line setting of HER2-positive breast cancer.

## 4 PD-1/L1 Inhibitors for Hormone Receptor-Positive/HER2-Negative Breast Cancer

Compared with other subtypes, hormone receptor-positive breast cancer is characterized as immunologically cold nature with lower PD-L1 expression, lower levels of TILs, and lower TMB (13, 64, 65). The efficacy of PD-1/PD-L1 inhibitor monotherapy in hormone receptor-positive metastatic breast cancer is limited (41, 66). Therefore, immune combination therapy is an approach to improve the efficacy of immunotherapy in hormone receptor-positive breast cancer. The I-SPY2 study included 52 hormone receptor-positive/HER2-negative patients (18). The combination of durvalumab, olaparib, and chemotherapy was promising for hormone receptor-positive breast cancer patients at high risk of MammaPrint, with a pCR of 28% compared with 14% in the chemotherapy-alone group (18). The phase II CheckMate7A8 (NCT04075604) (67) and phase III CheckMate7FL (NCT04109066) (68) about nivolumab combined with endocrine therapy in the neoadjuvant setting of hormone receptor-positive/HER2-negative breast cancer are enrolling. However, the addition of pembrolizumab to eribulin for metastatic hormone receptor-positive breast cancer patients who pretreated with 0 to 2 lines of salvage chemotherapy did not improve ORR, PFS, or OS (immature) compared with eribulin alone (69). Cyclin-dependent kinase (CDK) 4 and 6 inhibitors were demonstrated to be capable of increase levels of tumor-infiltrating T cells and yield synergic antitumor efficacy with anti-PD-1/L1 inhibitors in preclinical studies (70, 71). A phase Ib trial assessed the safety and antitumor activity of pembrolizumab plus abemaciclib in endocrine-resistant hormone receptor-positive/HER2-negative patients who were pretreated with 1 or 2 chemotherapy regimens for metastatic disease (72). The results showed that the ORR and DCR was 29% and 82%, respectively. Median PFS reached 8.9 months, and OS reached 26.3 months. Safety was generally tolerable and consistent with known side effects of individual drug.

## 5 Predictive Biomarkers of Efficacy

Current studies suggested that not all patients were sensitive to immunotherapy or immune combination therapy. Therefore, it is essential to explore biomarkers predictive of efficacy to screen beneficiary populations and avoid blind application of expensive but minimally effective agents. Some studies have shed some light on us about selecting sensitive subpopulation.

In SAFIR02-BREAST IMMUNO study, exploratory analyses identified *CD274* gene (encodes the CD274 molecule namely PD-L1) amplification as a potential biomarker of sensitivity to durvalumab (19); however, tumor infiltration lymphocytes (CD8, FoxP3, and CD103) and homologous recombination deficiency did not predict that (19). Exploratory efficacy analyses in IMpassion130 suggested that PD-L1 expressed on tumor-infiltrating immune cells is the most powerful biomarker for predicting survival benefits of immunotherapeutic regimen for patients with untreated advanced or metastatic TNBC (73). In the study by Schmid et al., AKT inhibitor plus atezolizumab and chemotherapy benefited TNBC patients irrespective of PD-L1 status and PIK3CA/AKT1/PTEN alteration status (74). Tolaney et al. found that PD-L1 detected by PD-L1 22C3 assay did not predict the efficacy of pembrolizumab in combination with eribulin in hormone receptor-positive patients (69). Notably, PD-L1 detection approaches differ in studies because of the differences in assays and interpretation standards, which leads to inconsistent PD-L1 prevalence. For example, the *post-hoc* analysis of IMpassion130 found that the PD-L1-positive percentage was 46% for SP142 assay, 81% for 22C3 assay, and 75% for SP263 assay (75). Standardization of detection assays is another challenge of precisely guiding the prescription of immunotherapy. Studies have shown that high tumor mutation burden (TMB) can predict the efficacy of breast cancer immunotherapy (76, 77), but there is no uniform standard for TMB threshold. Although there are some discoveries in biomarker exploration currently, and PD-L1 is recognized, it is still insufficient for tumor microenvironment and biomarker research. Future studies that identify biomarker-defined subgroups are needed to select breast cancer patients that could significantly benefit from immunotherapy.

## 6 Conclusions and Expectations

Immunotherapy has developed rapidly in the field of breast cancer, especially in the exploration of TNBC treatment. Immune combination therapy including immunotherapy combined with chemotherapy, targeted therapy, or immune agonists has shown good efficacy and tolerable safety, which is superior to ICI monotherapy. However, there is no consensus on the difference between PD-1/L1 antibodies, optimal partners for combined treatments, the effect of dosing sequence on efficacy, and how long immunotherapy should be administered. The identification of predictive biomarkers of efficacy requires further exploration. Although a correlation between PD-L1 expression level and efficacy has been illustrated in multiple studies, some studies observed that PD-L1-negative patients could also benefit from immunotherapy. There are considerable variations between subtypes (triple-negative vs. other subtypes) and disease settings (early-stage vs. advanced-stage). Overall, the earlier stage immunotherapy is dosed, the higher the response rate. In addition, studies on immunotherapy combined with radiotherapy or local ablation therapy are ongoing. Immunotherapy is promising in the treatment of various types of breast cancer.

## Author Contributions

FC carried out the primary literature search, drafted and revised the manuscript, and participated in discussions. NC, YG, LJ, and ZL helped modify the manuscript. JC carried out the literature analysis, drafted and revised the manuscript, and participated in discussions. All authors read and approved the final manuscript.

## Funding

This study was supported by the Key Laboratory Construction Project of Department of Science and Technology of Jilin Province (No. 20170622011JC) and the Special Project for Provincial Industrial Innovation of Development and Reform Commission in Jilin Province (No. 2017C022).

## Conflict of Interest

The authors declare that the research was conducted in the absence of any commercial or financial relationships that could be construed as a potential conflict of interest.

## Publisher’s Note

All claims expressed in this article are solely those of the authors and do not necessarily represent those of their affiliated organizations, or those of the publisher, the editors and the reviewers. Any product that may be evaluated in this article, or claim that may be made by its manufacturer, is not guaranteed or endorsed by the publisher.

## Supplementary Material

Supplementary Table 1 Main characteristics of cited studies. mTNBC, metastatic triple-negative breast cancer; pCR, pathological complete response; EFS, event-free survival; IDFS, invasive disease-free survival; PFS, progression-free survival; OS, overall survival; ORR, objective response rate; AEs, adverse events; DCR, disease control rate; m, months. *Durvalumab 2 weeks before the beginning of nab-paclitaxel.
