# Supplementary material for: Clinical Progress of PD-1/L1 Inhibitors in Breast Cancer Immunotherapy
Source: Front Oncol. 2022 Jan 6;11:724424. doi: 10.3389/fonc.2021.724424 (PMC8770281; doi:10.3389/fonc.2021.724424)
Supplement: Supplementary Table 1 — Main characteristics of cited studies. mTNBC, metastatic triple-negative breast cancer; pCR, pathological complete response; EFS, event-free survival; IDFS, invasive disease-free survival; PFS, progression-free survival; OS, overall survival; ORR, objective response rate; AEs, adverse events; DCR, disease control rate; m, months. *Durvalumab 2 weeks before the beginning of nab-paclitaxel. [file Table_1.docx]

| **Trial (phase, area)** | **Treatments arms (n)** | **Primary endpoint(s)** | | **median follow-up time** | **PFS (EFS for neoadjuvant therapy)** | | **OS** | **ORR (pCR for neoadjuvant therapy)** | **Overall grade ≥3 AEs** |
| --- | --- | --- | --- | --- | --- | --- | --- | --- | --- |
| **Triple-negative breast cancer** | | | | | | | | | |
| **Neoadjuvant therapy** | | | | | | | | | |
| KEYNOTE-522 (III, international) | pembrolizumab + carboplatin + paclitaxel and sequential doxorubicin/epirubicin + cyclophosphamide (784) vs placebo + carboplatin + paclitaxel and sequential doxorubicin/epirubicin + cyclophosphamide (390) | | pCR and EFS | 15.5m | 18-month EFS: 91.3% vs 85.3% | not reached | | overall population: 64.8% vs 51.2% | 78.0% vs 73.0% |
|  |  |  |  |  |  |  |  | PD-L1 positive group: 68.9% vs 54.9% |  |
|  |  |  |  |  |  |  |  | PD-L1 negative group: 45.3% vs 30.3% |  |
| NeoTRIPaPDL1 (III, international) | atezolizumab + carboplatin/nab-paclitaxel (138) vs placebo + carboplatin/nab-paclitaxel (142) | | EFS | not reported | not reached | not reached | | 43.5% vs 40.8% |  |
|  |  |  |  |  |  |  |  | PD-L1 positive group: 51.9% vs 48.0% |  |
| IMpassion031 (III, international) | atezolizumab + nab-paclitaxel + sequential doxorubicin/cyclophosphamide (165) vs placebo + nab-paclitaxel + sequential doxorubicin/cyclophosphamide (168) | | pCR | 20.6m vs 19.8m | not reached | not reached | | overall population: 57.6% vs 41.1% | 57%% vs 53% |
|  |  |  |  |  |  |  |  | PD-L1 positive group: 68.8% vs 49.3% |  |
|  |  |  |  |  |  |  |  | PD-L1 positive group: 69% vs 49% |  |
| GeparNuevo (II, Germany) | durvalumab + nab-paclitaxel (88) vs placebo + nab-paclitaxel (86) | | pCR | not reported | not reached | not reached | | window group*: 61.0% versus 41.4% | not reported |
|  |  |  |  |  |  |  |  | non-window group: 53.4% vs 44.2% |  |
| I-SPY2 (II,  United States) | durvalumab + olaparib + paclitaxel + sequential doxorubicin/cyclophosphamide  vs paclitaxel + sequential doxorubicin/cyclophosphamide (a total of 21 patients) | | pCR | not reported | not reached | not reached | | 47% vs 27% | 58% vs 41% (TNBC and HR+ patients) |
| **Adjuvant therapy** | | | | | | | | | |
| SWOG S1418/NRG BR006 (III, international) | pembrolizumab vs observation | | IDFS | ongoing | | | | | |
| IMpassion 030 (III, international) | atezolizumab + nab-paclitaxel vs placebo + nab-paclitaxel | | IDFS | ongoing | | | | | |
| **Maintenance therapy** | | | | | | | | | |
| SAFIR02-IMMUNO (II, France) | durvalumab (47) vs chemotherapy (35) | | PFS | not reported | not reported | | 21m vs 14m | not reported | 13.2% vs 15.9% (all subtypes patients) |
| **First-line** | | | | | | | | | |
| KEYNOTE-086-cohort B (II, international) | pembrolizumab 200 mg/3w for up to 2y (84) | | safety | 12.3 m | 2.1m | | 18m | 21.4% | 9.5% |
| PCD4989g (Ia, international) (first-line patients) | atezolizumab (21) | | safety and tolerability | 25.3m | not reported | | 17.6m | 24% | 21% (all patients) |
| IMpassion130 (III, international) | atezolizumab 840 mg on D1&15/4w + nab-paclitaxel (451) vs placebo + nab-paclitaxel (451) | | PFS & OS | 18.5m vs 17.5m | overall ITT population:  7.2m vs 5.5m (HR 0.80, 95% CI 0.69 - 0.92, *P* = 0.0021); 1-year PFS 24% vs 18% | | overall ITT population:  21m vs 18.7m (HR 0.86, 95% CI 0.72 - 1.02; *P* = 0.078); 2-year OS 42% vs 39% | overall ITT population:  56.0% vs 45.9% | 40.4% vs 30.7% |
|  |  |  |  |  | PD-L1 ≥1%:  7.5m vs 5.0m (HR 0.62, 95% CI 0.49 - 0.78, *P* < 0.0001), 1-year PFS 29% vs 16% | | PD-L1 ≥1%:  25m vs 18m (HR 0.71, 95% CI 0.54 - 0.94), 2-year OS 51% vs 37% | PD-L1 ≥1%:  58.9% vs 42.6% |  |
| IMpassion131 (III, international) | atezolizumab 840 mg on D1&15/4w + paclitaxel vs placebo + paclitaxel (651 patients were allocated with a 2:1 ratio) | | PFS | not reported | no significant difference (news release from Roche Company) | | not reached | not reported | not reported |
| IMpassion132 (III, international) | atezolizumab 1200 mg/3w + gemcitabine/carboplatin or capecitabine vs placebo + gemcitabine/carboplatin or capecitabine | | OS | ongoing | | | | | |

| KEYNOTE-355 (III, international) | pembrolizumab 10 mg/kg/2w + nab-paclitaxel or paclitaxel or gemcitabine/carboplatin (566) vs placebo + nab-paclitaxel or paclitaxel or gemcitabine/carboplatin (281) | PFS and OS | 17.5m vs 15.5m | overall ITT population:  7.5m vs 5.6m (HR 0.82, 95% CI 0.69-0.97) | not reached | not reported | 68.1% vs 66.9% |
| --- | --- | --- | --- | --- | --- | --- | --- |
|  |  |  |  | PD-L1 ≥ 1%:  7.6m vs 5.6m (HR 0.74, 95% CI  0.61-0.90, *P* = 0.0014 > 0.00111) |  |  |  |
|  |  |  |  | PD-L1 ≥10%:  9.7m vs 5.6m (HR 0.65, 95% CI 0.49-0.86, *P* = 0.0012 < 0.00411); 1-year PFS 39.1% vs 23% |  |  |  |

| NCT03800836 (Ib, international) | atezolizumab 840 mg on D1&15/4w + ipatasertib + paclitaxel or nab-paclitaxel (26) | ORR | 6.1m | not reported | not reported | overall ITT population: 73% | | 54% |
| --- | --- | --- | --- | --- | --- | --- | --- | --- |
|  |  |  |  |  |  | PD-L1 ≥1%: 82% | |  |
|  |  |  |  |  |  | PD-L1 < 1%: 75% | |  |
| WJOG9917B NEWBEAT (II, Japan) | nivolumab 240 mg on D1&15/4w + bevacizumab + paclitaxel (32% TNBC, n = 18; 68% HR+ breast cancer, n = 39) | ORR | 10.7m | not reached | not reached | TNBC population: 83.3% | | 58% (overall population) |
|  |  |  |  |  |  | HR+ population: 71.7% | |  |
| NCT03394287 (II, China) | camrelizumab 200mg/2w + apatinib 250mg continuous dosing [D1-14] (24) or intermittent dosing [D1-7] (10) | ORR | not reported | continuous dosing group: not reached | not reported | continuous dosing group: 47.4% | | not reported |
|  |  |  |  | intermittent dosing group: 2m |  | intermittent dosing group: 0% | |  |
| COLET (II, international) | atezolizumab + cobimetinib + nab-paclitaxel or paclitaxel (63) | ORR | 6.5m | not reported | not reported | nab-paclitaxel group: 29% | | nab-paclitaxel group: 70% |
|  |  |  |  |  |  | paclitaxel group: 34% | | paclitaxel group: 69% |
| TONIC (II, Netherlands) | radiotherapy, doxorubicin, cyclophosphamide, and cisplatin + sequential nivolumab (66) | PFS | not reported | not reported | not reported | 23% | | not reported |
| **Second-line or later therapy** | | | | | | | | |
| KEYNOTE-012 (Ib, international) | pembrolizumab 10 mg/kg/2w (32) | ORR | 10.7m | 1.9m; 1-year PFS: 15.0% | 10.2m; 1-year OS: 41.1% | 18.5% | | 18.8% |
| KEYNOTE-086-cohort A (II, international) | pembrolizumab 200 mg/3w for up to 2y for previously treated mTNBC (32) | ORR | 9.6m | Overall population: 2.0m | Overall population: 9.0m | Overall population: 5.3% | | Overall population: 12.9% |
|  |  |  |  | PD-L1-positive population: 2.0m | PD-L1-positive population: 8.8m | PD-L1-positive population: 5.7% | |  |
| KEYNOTE-119 (III, international) | pembrolizumab 200 mg/3w (312) vs capecitabine, eribulin, gemcitabine, or vinorelbine (310) | OS | 9.9m vs 11.2m | overall ITT population:  2.1m vs 3.3m (HR 1.60, 95% CI 1.33-1.92) | overall ITT population:  9.9m vs 10.8m (HR 0.97, 95% CI 0.82 – 1.15) | overall ITT population:  9.6% vs 10.6% | | 14% (1 death) vs 36% (2 deaths) |
|  |  |  |  | PD-L1 ≥1% population: 2.1m vs 3.1m (HR 1.35, 95%CI 1.08 – 1.68) | PD-L1 ≥1% population: 10.7m vs 10.2m (HR 0.86, 95%CI 0.69 – 1.06) | PD-L1 ≥1% population: 12.3% vs 9.4% | |  |
|  |  |  |  | PD-L1 ≥10% population: 2.1m vs 3.4m (HR 1.14, 95%CI 0.82 – 1.59) | PD-L1 ≥10% population: 12.7m vs 11.6m (HR 0.78, 95%CI 0.57 – 1.06) | PD-L1 ≥10% population: 17.7% vs 9.2% | |  |
|  |  |  |  |  | PD-L1 ≥20% population: 14.9m vs 12.5m (HR 0.58, 95%CI 0.38 – 0.88) |  |  |  |
| JAVELIN (1b, international) | avelumab 10 mg/kg/2w (58) |  | not reported | not reported | not reported | PD-L1 non-selected patients: 5.2% | | not reported |
|  |  |  |  |  |  | PD-L1≥1% population: 22.2% | |  |
| PCD4989g (1a, international) (second-line and beyond patients) | atezolizumab (95) | safety and tolerability | 25.3m | not reported | 7.3m | 11% | | 21% (all patients) |
| TOPACIO/KEYNOTE-162 (II, United States) | pembrolizumab + niraparib (55) | ORR | 12.4m | BRCA mutation patients: 8.3m | not reported | overall patients: 21% | | 58% |
|  |  |  |  | wild-type BRCA patients: 2.1m |  | BRCA mutation patients: 47% | wild-type BRCA patients: 11% |  |
|  |  |  |  |  |  | PD-L1-positive patients: 32% | PD-L1-negative patients: 8% |  |
| MEDIOLA (I/II, international) | durvalumab + olaparib (34) | safety, tolerability, and 12-week DCR | 6.7 m | 8.2 m | 21.5 m | 63.3% | | 33% |
| TONIC (II, Netherlands) | radiotherapy, doxorubicin, cyclophosphamide, and cisplatin + sequential nivolumab (66) | PFS | not reported | not reported | not reported | second-line: 45%  third-line or more lines: 32% | | not reported |
| IMPRIME1 (II, United States) | pembrolizumab + Imprime PGG (44) | ORR and safety | not reported | 16.4m | overall patients: 12-month OS: 57.6%; 18-month OS: 36.7% | overall patients: 15.9% | | 6.8% |
|  |  |  |  |  | previous HR+ patients: 17.1m | previous HR+ patients: 50% | |  |
| **HER2 positive breast cancer** | | | | | | | | |
| **Second-line and later therapy** | | | | | | | | |
| CCTG IND.229 (Ib,  Canada) | durvalumab + trastuzumab (15) | recommended phase II dose | not reported | 1.35m; estimated 6‐month PFS: 0% | estimated 6‐month OS: 51.6%, 1‐year OS: 17.2% | 0% | | not reported |
| PANACEA (Ib-II, international) | pembrolizumab + trastuzumab (58) | the proportion of PD-L1-positive patients achieving an objective response. | PD-L1-positive patients: 13.6m | PD-L1-positive patients: 2.7m | PD-L1-positive: not reached | PD-L1-positive patients: 15% | | 29% |
|  |  |  | PD-L1-negative patients: 12.2m | PD-L1-negative patients: 2.5m | PD-L1-negative: 7.0m | PD-L1-negative patients: 0% | |  |
| KATE2 (II, international) | atezolizumab+ T-DM1 (133) vs T-DM1 + placebo (69) | PFS and safety | 8.5m vs 8.4m | overall patients: 8.2m vs 6.8m | not reached | overall patients: 45% vs 43% | | not reported |
|  |  |  |  | PD-L1-positive patients: 8.5m vs 4.1m |  | PD-L1-positive patients: 54% vs 33% | |  |
|  |  |  |  | PD-L1-negative patients: 6.8m vs 8.2m |  | PD-L1-negative patients: 39% vs 50% | |  |
| NCT03032107 (Ib, United States) | pembrolizumab + T-DM1 (20) | safety and tolerability | 23.5m | 9.6m | not reached | 20% | | 20% |
| **HR-positive, HER2 negative breast cancer** | | | | | | | | |
| **Neoadjuvant therapy** | | | | | | | | |
| I-SPY2 (II,  United States) | durvalumab + olaparib + paclitaxel + sequential doxorubicin/cyclophosphamide  vs paclitaxel + sequential doxorubicin/cyclophosphamide (a total of 52 patients) | pCR | not reported | not reached | not reached | 28% vs 14% | | 58% vs 41% (TNBC and HR+ patients) |
| CheckMate7A8 (II, international) | nivolumab + abemaciclib or palbociclib + anastrozole | safety and residual cancer burden 0-1 rate | ongoing | | | | | |
| CheckMate7FL (III, international) | nivolumab + neoadjuvant chemotherapy and adjuvant endocrine therapy vs placebo + neoadjuvant chemotherapy and adjuvant endocrine therapy | pCR and EFS | ongoing | | | | | |
| **Second-line and later therapy** | | | | | | | | |
| NCT02779751 (Ib, international) | pembrolizumab + abemaciclib (28) | safety | not reported | 8.9m | 26.3m | 29% | | not reported |
| mTNBC: metastatic triple-negative breast cancer; pCR: pathological complete response; EFS: event-free survival; IDFS: invasive disease-free survival; PFS: progression free survival; OS: overall survival; ORR: objective response rate; AEs: adverse events; DCR: disease control rate; *: durvalumab 2 weeks before the beginning of nab-paclitaxel; m: months. | | | | | | | | |
